# Safety of Aflibercept in Metastatic Colorectal Cancer: A Literature Review and Expert Perspective on Clinical and Real-World Data

**DOI:** 10.3390/cancers12040844

**Published:** 2020-03-31

**Authors:** Kei Muro, Taylor Salinardi, Arvind Rup Singh, Teresa Macarulla

**Affiliations:** 1 Department of Clinical Oncology, Aichi Cancer Center Hospital, Nagoya 464-8681, Japan; 2 Global Medical Oncology, Sanofi, Boston, MA 02142, USA; Taylor.Salinardi@sanofi.com (T.S.); Arvind.Singh@sanofi.com (A.R.S.); 3 Gastrointestinal Tumors Service of the Medical Oncology Service, Vall d’Hebron University Hospital, Vall d’Hebron Institute of Oncology, IOB, Barcelona 08035, Spain; tmacarulla@vhio.net

**Keywords:** colorectal cancer, mCRC, VEGF inhibitor, anti-angiogenic therapy, aflibercept, clinical practice

## Abstract

Background: Metastatic colorectal cancer (mCRC) represents a substantial health burden globally and an increasing challenge in Asian countries. Treatment options include chemotherapy plus a vascular endothelial growth factor (VEGF) inhibitor (such as bevacizumab, aflibercept or ramucirumab), or anti-epidermal growth factor receptor (EGFR) therapies. Aflibercept, a recombinant fusion protein, has been approved for treatment of mCRC in combination with FOLFIRI for patients whose disease progresses during or after treatment with an oxaliplatin-containing regimen, based on its efficacy and tolerability profile in clinical trials. This report aims to provide an overview of both clinical and real-world evidence and experience on the use of aflibercept in routine clinical practice, with a focus on European, American and Asian populations. Methods: A literature search was conducted in PubMed (on 28th February 2019) using the search terms ("aflibercept") and ("Colorectal"OR"CRC") to identify publications containing information on aflibercept-containing regimens. Results: The adverse events (AE) profile was similar between geographical locations. Across trials, real-world and retrospective studies, grade ≥ 3 hypertension and proteinuria were amongst the most frequently reported AEs. Conclusions: The safety profile of aflibercept is generally manageable and comparable across various geographic locations.

## 1. Introduction

The global burden of colorectal cancer (CRC) is expected to increase by 60% to more than 2.2 million new cases and 1.1 million deaths by 2030. It is the second most frequently diagnosed cancer and the fourth leading cause of cancer-related death worldwide [1,2]). In Europe, 2018 estimates indicate that CRC is the second most frequently occurring cancer and cause of death from cancer [3]. Increases in incidence and mortality have been seen in Eastern Europe, where as other European countries have exhibited an increase in incidence but a decline in mortality [1]. In Asia, CRC rates are increasing rapidly, and it is now the third most frequently occurring malignancy in both men and women. Mortality from CRC has been increasing in Asian countries over the last decade, with the exception of Japan and Singapore [1,4]. According to the vital statistics of Japan in 2015, CRC was the most frequent cause of cancer deaths in women [5]. A multinational cohort study involving four Asian countries (Taiwan, Korea, Japan, and Hong Kong) demonstrated that the incidence of young-onset CRC has significantly increased in both men and women in the past two decades [6]. Although CRC occurrence is similar in Europe and Asia, the treatment strategies are different. 

This paper aims to evaluate current safety data reported for aflibercept in mCRC using clinical and real-world/retrospective data. After reviewing these data, insights on patient management from different perspectives will be provided in order to help inform treatment decisions in this disease setting.

## 2. Methods

**Figure 1:**
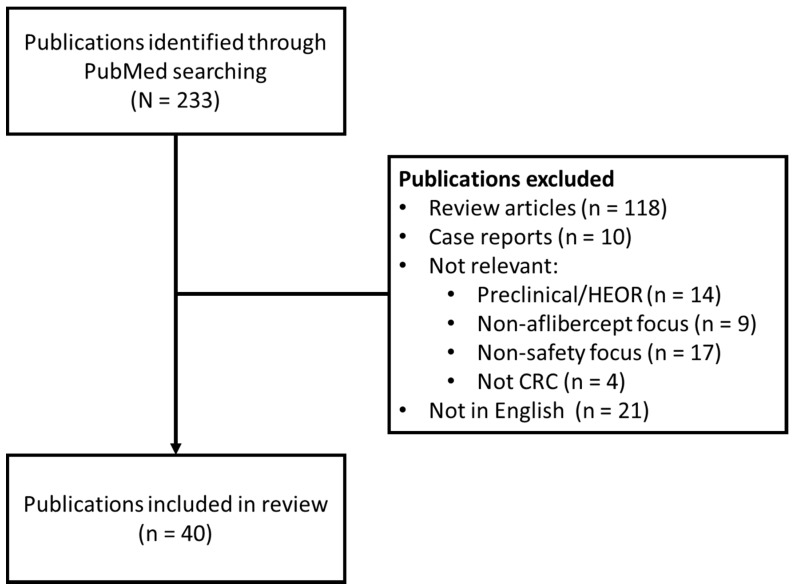
PRISMA flow diagram: summary of literature search outcomes.

A literature search was conducted in PubMed using the search terms (“aflibercept”) and (“colorectal”OR“CRC”) to identify publications available cumulatively until March 13, 2020 containing information on aflibercept-containing regimens. Identified publications were reviewed and those including safety data were selected. Both clinical trials and real-world data (including observational and retrospective studies) were included. Review articles and reports with a focus on efficacy were excluded from the analysis. In order to obtain studies reporting safety data from a sufficient number of patients, individual case reports/case series with < 5 patients were also excluded. Google Scholar was used to access an article known to have only been presented at a congress (Figure 1).

## 3. Results

### Aflibercept Mechanism of Action and Relevance To Safety Profile

**Figure 2:**
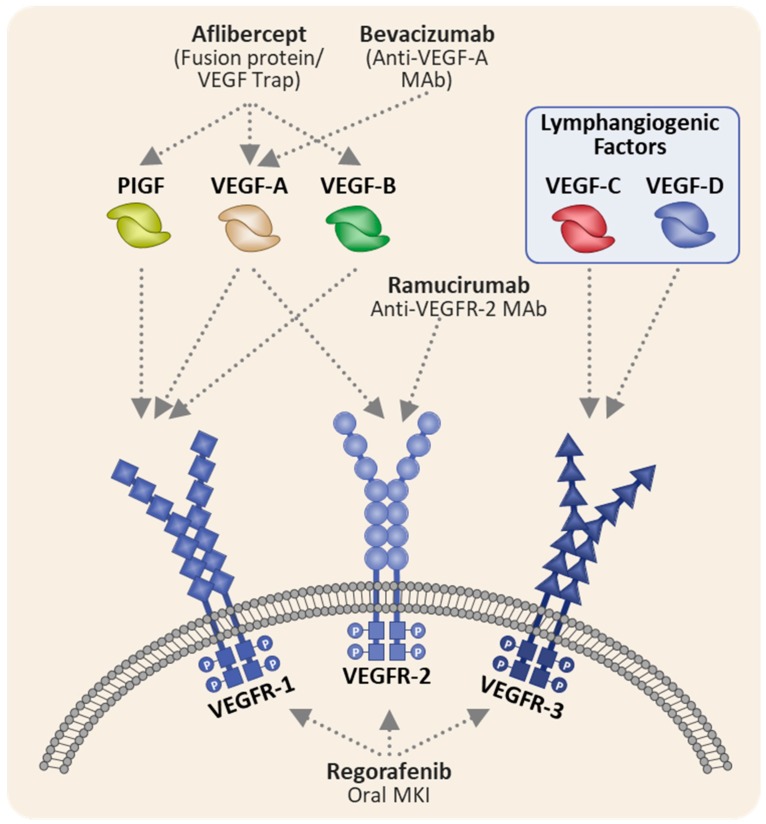
Mechanism of action of anti-VEGF agents in colorectal cancer (adapted from Clarke et al. [8]). mAB, monoclonal antibody; MKI, multikinase inhibitor; PlGF, placental growth factor; VEGF, vascular endothelial growth factor; VEGF-R, vascular endothelial growth factor receptor. VEGFR-1 is transcribed from the Fms-related receptor tyrosine kinase (FLT1) gene, VEGFR-2 from the Kinase Insert Domain Receptor (KDR) and VEGFR-3 from the FLT3 gene.

Aflibercept is a recombinant fusion protein composed of the second immunoglobulin (Ig)-like domain of FLT1 (VEGFR1) joined to the third Ig-like domain of KDR (VEGFR2) fused to the Fc portion of human IgG1 (Figure 2). Aflibercept differs from other anti-VEGF agents by functioning as a decoy receptor with high-affinity for other angiogenic factors, VEGF-B and placental growth factor (PlGF). Additionally, PlGF has been proposed as a mechanism of bevacizumab resistance. Consequently, aflibercept’s binding affinity for PlGF has been hypothesized to contribute to its efficacy in the post-bevacizumab setting [7].

Tumors exploit angiogenic pathways to facilitate rapid and uncontrolled proliferation. Using anti-angiogenics inhibits the formation of new vessels within the tumor and leads to hypoxia and tumor death. Although angiogenesis occurs at a lower rate in adults, these pathways are used for vascular homeostasis. For example, VEGF promotes the release of nitric oxide, a vasodilator, which may explain why anti-angiogenics are frequently associated with hypertension. Proteinuria, another frequent adverse event (AE) associated with anti-angiogenics, may be due to VEGF reductions leading to decreased nephrin expression, subsequently resulting in podocyte dysfunction. Anti-angiogenics are also associated with non-cardiovascular AEs, due to the complexity of the VEGF signaling pathway [9,10].

## 4. Safety Profile of Aflibercept-Containing Therapy in mCRC

### 4.1. Aflibercept and FOLFOX/FOLFIRI in Clinical Trials

#### 4.1.1. Global Studies

**Figure 3:**
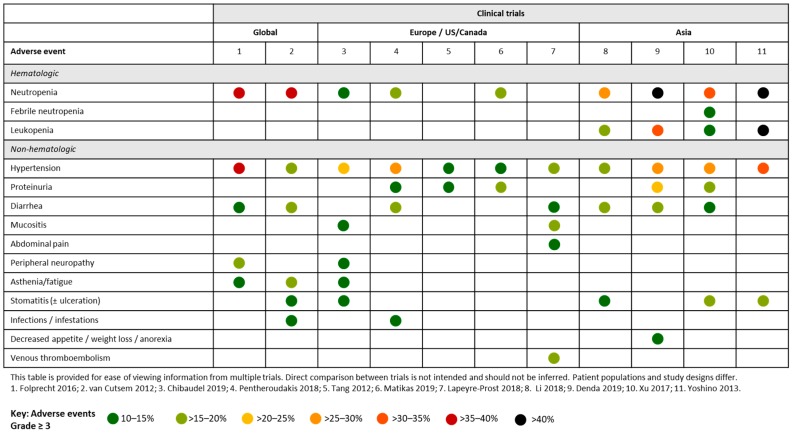
Summary of Grade ≥ 3 adverse events occurring in ≥ 10% of patients across clinical trials of aflibercept in patients with mCRC.

The safety and efficacy of aflibercept has been evaluated in several global clinical trials (Figure 3). VELOUR was an international randomized, placebo-controlled Phase III trial comparing the efficacy and safety of aflibercept plus FOLFIRI (*n* = 612) and placebo plus FOLFIRI (*n* = 614) in patients with mCRC who had previously received oxaliplatin based chemotherapy. Addition of aflibercept to FOLFIRI significantly improved efficacy outcomes compared with placebo plus FOLFIRI. 

The experimental arm demonstrated a statistically significant benefit in overall survival (OS; median 13.50 months versus 12.06 months; *p* = 0.0032), progression-free survival (PFS; median 6.90 months versus 4.67 months; *p* < 0.0001), and response rates (19.8% versus 11.1%; *p* = 0.0001) compared with the control arm. The most frequently occurring Grade ≥ 3 AEs in patients who received aflibercept plus FOLFIRI were neutropenia (laboratory measurements: 36.7%, versus 29.5% for placebo plus FOLFIRI), hypertension (19.3%, versus 1.5% for placebo plus FOLFIRI), diarrhea (19.3%, versus 7.8% for placebo plus FOLFIRI), asthenia (16.8%, versus 10.6% for placebo plus FOLFIRI), stomatitis and ulceration (13.8%, versus 5.0% for placebo plus FOLFIRI) and infections and infestations (12.3%, versus 6.9% for placebo plus FOLFIRI). AEs observed at ≥ 5% in either arm are summarized in Table 1. Rates of any grade arterial thrombotic events were 1.5% for placebo plus FOLFIRI (0.5% grade 3) and 2.6% for aflibercept plus FOLFIRI (0.8% grade 3 and 1.0% grade 4) [11]. In a post hoc analysis, the incidence of grade 3/4 AEs was higher for patients ≥ 65 years of age than for those < 65 years of age in both the aflibercept (89.3% vs 80.5%) and placebo (67.4% vs 59.4%) arms. Interaction tests for grade 3/4 antiangiogenic agent-related AEs suggested no heterogeneity between the older and younger patient populations (*p* > 0.1) [12]. Similarly, another post hoc analysis did not identify any differences in all grade and grade 3/4 AEs between the subgroup of patients with better and poorer efficacy [13]. Importantly, the majority of grade 3/4 AEs occurred within 4 cycles of treatment, in a small percent of treatment cycles, and were mostly reversible [14].

The Aflibercept Safety and Quality-of-Life Program (ASQoP) was an open-label single-arm trial evaluating the effect of second-line aflibercept plus FOLFIRI on the quality of life and safety of patients with mCRC in a real-world setting (in a similar patient population as the VELOUR study). An interim analysis based on 779 patients demonstrated that aflibercept-related AEs were similar in ASQoP and VELOUR, though the frequency of some AEs appeared lower in ASQoP, which may reflect more flexible *n* FOLFIRI dosing, or more experience in AE management (Table 2; [15,16]). 

The AFFIRM study compared aflibercept in combination with modified FOLFOX6 (mFOLFOX6; *n* = 119) with mFOLFOX6 alone (*n* = 117) for the first-line treatment of mCRC (Figure 3). Rates of AEs were generally increased in the aflibercept arm compared with mFOLFOX6 alone, and the most frequently occurring grade ≥ 3 AEs were neutropenia (36.1% vs 29.3%), hypertension (35.3% vs 1.7%), peripheral sensory neuropathy (16.8% vs 17.2%), and diarrhea (13.4% vs 5.2%). One patient died as a result of an intracranial hemorrhage (unrelated to aflibercept) and four others from infections (also determined not related to study treatment) [17]. In a separate analysis of the AFFIRM study data, no association was found between certain biomarkers (somatic mutations in oncogenic drivers of mCRC, common single-nucleotide polymorphisms in VEGF pathway genes and plasma markers) and AEs [18].

#### 4.1.2. European and US Studies

First-line aflibercept was evaluated in two European studies. In a French study, 49 patients with previously untreated mCRC received aflibercept plus FOLFOX followed by maintenance therapy with fluoropyrimidine (Figure 3). The most frequently occurring grade 3/4 AEs were hypertension (23%), fatigue (15%), neutropenia (laboratory measurement: 12%), neuropathy (12%) and stomatitis (10%). There were three (6%) treatment-related deaths due to stroke, pulmonary embolism and neutropenic sepsis [19]. In a study by the Hellenic Cooperative Oncology Group, first-line aflibercept plus FOLFIRI was evaluated in 73 patients with mCRC (Figure 3). The most frequently occurring grade 3/4 AEs were hypertension (26%), neutropenia (18%), diarrhea (15%), proteinuria (11%) and infections (11%). No deaths due to AEs were reported [20].

A Phase II study evaluated first-line FOLFIRI/aflibercept in Greek patients (N = 31) with mCRC. There was one toxic death due to sepsis. Grade 3/4 AEs included neutropenia (16.1%), diarrhea (19.4%), hypertension (12.9%), asthenia (9.7%), proteinuria (3.2%) and bowel perforation (3.2%) [21].

A Phase II single-arm multicentric study analyzed 40 patients with mCRC across nine French centers who were treated with first-line FOLFIRI. Grade 3/4 AEs were mainly gastrointestinal (45%: mucositis (15%), diarrhea (12.5%), abdominal pain (10%) and vascular (32.5%: hypertension (17.5%) and venous thromboembolism [15%]). Severe hematological toxicities occurred in 7.5% of patients. 87.5% of patients had at least one dose modification during treatment [22].

A Phase II study evaluated aflibercept in patients with mCRC, in Canada and the US, who had received prior bevacizumab (*n* = 51) or who were bevacizumab-naïve (*n* = 24) (Figure 3). The most frequently occurring grade 3/4 AEs were hypertension (13.5%) and proteinuria (10.8%). Ten patients discontinued aflibercept due to AEs, including five patients for severe hypertension and proteinuria [23].

#### 4.1.3. Asian Studies

Several clinical trials evaluating second-line aflibercept have been undertaken across Asia. A Phase III study of aflibercept plus FOLFIRI in Asian patients with pre-treated mCRC included 111 evaluable patients **(**Figure 3). The most frequently occurring grade ≥ 3 AEs were neutropenia (29%), hypertension (18%), diarrhea (17%) and stomatitis (11%). There were two treatment-related deaths in this patient group, four in the aflibercept plus FOLFIRI group and one in the placebo plus FOLFIRI only group [24].

In a Japan-specific study, 62 patients with mCRC were evaluated following second-line aflibercept plus FOLFIRI. The most frequently occurring grade 3/4 AEs were neutropenia (61.3%), hypertension (27.4%), diarrhea (19.4%) and decreased appetite (12.9%). There were no treatment-related deaths [25]. In a separate Japanese study, evaluating aflibercept (2 mg/kg or 4 mg/kg) plus FOLFIRI in 14 patients with previously treated mCRC, the most frequently occurring grade 3/4 AEs were neutropenia (76.9%), leukopenia (61.5%), hypertension (30.8%) and stomatitis (15.4%) [26].

A Phase I study has investigated aflibercept plus FOLFIRI in Chinese patients with metastatic solid malignancies (N = 20). In patients with mCRC (N = 15), the most common grade ≥ 3 AEs were neutropenia (35%), hypertension (30%), stomatitis (20%), proteinuria (20%), leukopenia (15%), febrile neutropenia (15%) and diarrhea (15%). There were no deaths during the study period [27].

Aflibercept had a consistent safety profile across clinical studies and regions. The most frequent AEs included neutropenia, hypertension and gastrointestinal issues. 

### 4.2. Aflibercept and FOLFOX/FOLFIRI in Real-World and Retrospective Studies

#### 4.2.1. European and the US Studies

**Figure 4:**
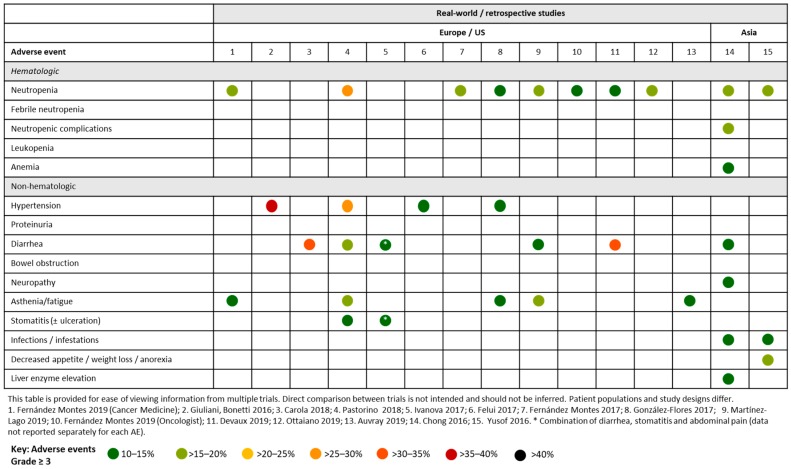
Summary of Grade ≥ 3 adverse events occurring in ≥ 10% of patients across real-world or retrospective studies of aflibercept in patients with mCRC.

Several retrospective studies have evaluated the real-world safety implication of aflibercept plus chemotherapy. In general, grade ≥ 3 AEs occurred at a lower rate compared with the rates observed in clinical trials (Figure 4).

Several retrospective and real-world studies have been performed to analyze Spanish patients (Figure 4). In one study, 167 patients were observed across seven hospitals. The main grade 3 toxicities associated with aflibercept plus FOLFIRI were neutropenia (16.2%), fatigue (6%) and diarrhea (5.4%). Grade ≥ 3 hypertension and thromboembolic events were observed in 7.2% and 6% of patients, respectively [28]. In another Spanish study, which evaluated 66 patients with mCRC who received aflibercept plus FOLFIRI, the most frequent grade 3/4 toxicities were asthenia (13%), neutropenia (11.5%), hypertension (10%), diarrhea (8%), stomatitis (3.2%), palmar-plantar erythrodysesthesia (3.2%) and proteinuria (5%) [29]. In another study evaluating 78 patients who received second-line aflibercept plus FOLFIRI, the most frequently occurring grade 3/4 toxicities were neutropenia (15.3%), asthenia (10.3%), diarrhea (6.4%) and mucositis (6.4%) [30]. Neutropenia (7.9%) was also the most frequently occurring grade ≥ 3 toxicity reported in a retrospective analysis of a Spanish Named Patient Program (NPP), which evaluated patients with mCRC receiving second-line aflibercept plus FOLFIRI. Other frequently reported grade ≥ 3 toxicities included gastrointestinal disorders (5.6%; mainly diarrhea, 4.5%) and vascular disorders (5.6%; mainly hypertension, 3.4%). Most Grade ≥ 3 AEs were reported during the initial treatment cycles. There were two treatment-related deaths that were due to intestinal perforation [31]. In another retrospective review of a Spanish NPP registry, where 71 patients with mCRC receiving aflibercept plus FOLFIRI were assessed, the most frequently reported grade 3 toxicities were hypertension (11.3%), neutropenia (9.9%), asthenia (8.5%) and diarrhea (8.5%) [32]. A retrospective observational study evaluated 71 elderly patients (> 70 years) with mCRC across seven hospitals from the Galician Research Group on Digestive Tumours. The most frequently occurring grade 3/4 AEs were asthenia (18.3%), neutropenia (15.5%), diarrhea (11.3%) and mucositis (9.9%). The most frequent grade 3/4 related toxicities associated with aflibercept were hypertension (5.6%), dysphonia (5.6%), proteinuria (2.8%). Two patients experienced grade 5 toxicity [33]. A real-world analysis of 250 Spanish patients with mCRC treated with aflibercept and irinotecan-based chemotherapy has been conducted. The most common grade 3/4 toxicities were neutropenia (13.4%), hypertension (6.8%), asthenia (6.8%), diarrhea (5.4%) and venous thrombotic disease (4.2%). Treatment-related mortality was 1.6% [34]. A real-world observational study evaluating 120 patients with RAS-WT mCRC who received second-line aflibercept plus FOLFIRI reported hypertension (7.5%), asthenia (5.9%) and perforation (2.5%) as the most frequently occurring [35].

In a French study evaluating FOLFIRI3-aflibercept in 65 evaluable patients with previously treated mCRC, the most frequently reported grade 3/4 AEs were diarrhea (30.8%), neutropenia (7.7%), hypertension (3.2%) and stomatitis (9.2%) (Figure 4) [36]. One monocentric retrospective study evaluated the FOLFIRI3 regimen given alone (*n* = 18) or in combination with bevacizumab (*n* = 99) or aflibercept (*n* = 36) in French patients with previously treated mCRC. The most frequent grade 3/4 AEs in the aflibercept plus FOLFIRI3 were diarrhea (33%) and neutropenia (14%) [37]. The AGEO study evaluated aflibercept plus chemotherapy beyond second-line therapy in patients with mCRC. The most frequent grade 3/4 AEs were asthenia (14.6%) and diarrhea (8.5%) [38].

An Italian study of 781 patients who received aflibercept plus FOLFIRI in the real-world setting reported a higher rate of AEs versus other studies (Figure 4). Hypertension (28.5%), neutropenia (27.5%; from laboratory data), asthenic conditions (20.0%), diarrhea (17.0%), and stomatitis (13.0%) were the most frequent grade 3/4 toxicities. One toxic death occurred during the study period due to sepsis, without neutropenic complications. There was no significant worsening of HRQL during treatment [39]. In a smaller Italian study of 74 patients with mCRC treated with either second-line FOLFIRI/bevacizumab (Arm A; *n* = 31) or FOLFIRI/aflibercept (Arm B; *n* = 43), the most frequently occurring grade 3 toxicity was neutropenia (16.3% in Arm B). Grade 3 cardiovascular events occurred in 7.0% of patients in Arm B. No grade 4 events were observed [40]. 

QoLiTrap was a multinational non-interventional study evaluating the quality of life of 1500 German patients with mCRC previously treated with FOLFIRI plus aflibercept (Figure 4). The most frequent AEs were diarrhea, oral mucositis, fatigue, nausea, and hypertension, predominantly grade ≤ 2 [41].

In a US study of aflibercept-containing therapy as a second-line or later-line treatment for mCRC (N = 218), the most frequent grade ≥ 3 AEs were gastrointestinal disorders (11.0%), asthenia/fatigue (8.7%), neutropenia (8.7%) and hypertension (6.4%) (Figure 4) [42].

#### 4.2.2. Asian studies

In a retrospective analysis of 19 patients with mCRC (84% of Chinese ethnicity) who received second-line aflibercept plus FOLFIRI via a NPP, the most frequently reported grade 3 toxicities were neutropenia (15.8%), neutropenic complications (15.8%), diarrhea (10.5%), bowel obstruction (10.5%), anemia (10.5%) and liver enzyme elevation (10.5%). There were no grade 4 AEs or treatment-related deaths, and all AEs resolved with supportive care management [43] (Figure 4).

Yusof and colleagues conducted a retrospective analysis of Malaysian patients (N = 25) receiving first-line (*n* = 3) or second-line (*n* = 22) aflibercept plus FOLFIRI via a NPP. The most frequently occurring grade 3/4 AEs were weight loss/anorexia (20%), neutropenia (16%), infection (12%), hypertension (8%) and febrile neutropenia (8%). AEs were generally reversible and manageable and consistent with those observed in Western populations [44] (Figure 4).

A retrospective study conducted in Taiwan evaluated aflibercept monotherapy in patients with mCRC (N = 15) who had refractory ascites failing to respond to standard chemotherapy. No drug-related AEs were reported; however only a small number of patients were evaluated [45]. 

Two studies assessing aflibercept plus FOLFIRI in 62 patients and 16 patients with mCRC, respectively, have reported a higher incidence of hematologic AEs compared with other larger studies [25,26].

Overall, safety profiles reported in registry and real-world studies were consistent with those reported in clinical trials. However, rates of grade ≥ 3 AEs occurred were lower compared with those observed in clinical trials.

## 5. Management of aflibercept-Associated Adverse Events in mCRC

AEs frequently associated with aflibercept therapy for CRC, irrespective of severity, include neutropenia, leukopenia, proteinuria, hypertension, diarrhea, stomatitis, fatigue, decreased weight, decreased appetite, elevated liver enzymes, abdominal pain, dysphonia, increased serum creatinine and headache. Severe AEs have included serious or fatal risk of bleeding, gastrointestinal perforation, and delayed wound healing, though some are related to chemotherapy (generally FOLFORI) [46,47,48].

The precise mechanisms responsible for the toxicities of aflibercept are not yet fully elucidated. However, hypertension may be influenced by VEGF interaction with the angiotensin system, and proteinuria may be related to VEGF effects on the renal glomerulus [47,48]. Thrombosis and hemorrhage could be related to the role of VEGF in vascular integrity [48]. Recommendations for managing the most clinically-relevant AEs associated with aflibercept therapy have been published and focus on hypertension, proteinuria, gastrointestinal perforation, thromboembolism and hemorrhage [48]. These are summarized in Table 3. 

### Management of hypertension and proteinuria

Across trials, real-world and retrospective studies, grade ≥ 3 hypertension and proteinuria are among the most frequently reported AEs. Aflibercept prescribing information contains guidance for timely identification and management of hypertension. Patients receiving aflibercept should have their blood pressure monitored at least every 2 weeks or more regularly as clinically indicated throughout treatment and hypertension should be actively controlled with appropriate therapy. Aflibercept should be withheld in patients whose hypertension is not adequately controlled and only resumed at a reduced dose (2 mg/kg) for subsequent cycles once hypertension is controlled. Aflibercept should be permanently discontinued for development of hypertensive crisis or hypertensive encephalopathy (Aflibercept PI, 2016). Note that there is no clinical trial experience with aflibercept in patients with New York Heart Association (NYHA) class III/IV heart failure, as it was an exclusion criterion.

There is also specific guidance for management of proteinuria and patients should be monitored by urine dipstick analysis and/or urinary protein creatinine ratio (UPCR) during aflibercept therapy. In case of dipstick ≥ 2+ or UPCR > 1, 24-h urine collection is mandated. Aflibercept should be withheld for proteinuria ≥ 2 g/24 h and only resumed when resolved to < 2 g/24 h Aflibercept should be suspended for recurrent proteinuria and resumed at a reduced dose (2 mg/kg) on resolution. Aflibercept should be permanently discontinued for development of nephrotic syndrome or thrombotic microangiopathy [48].

## 6. Management of Aflibercept-Related Adverse Events in My mCRC Patients—A European Perspective

The European Society for Medical Oncology (ESMO) guidelines provide clinical recommendations for the management of mCRC and are implemented routinely in European clinical practice, though not for the management of treatment-related AEs [49].

In my experience, the most frequently occurring AEs in patients who receive aflibercept are hypertension and proteinuria. Management of hypertension is achieved through anti-hypertensive medication, effective patient education and active monitoring throughout treatment. If blood pressure is not controlled by anti-hypertensive medication, temporary discontinuation of aflibercept is considered. Importantly, patients with a persistent blood pressure of ≥ 150/90 are instructed to contact the emergency room for immediate treatment. Proteinuria is effectively managed by regular monitoring by dipstick test throughout treatment and temporary treatment discontinuation.

The frequency and intensity of AEs related to chemotherapy (FOLFIRI) can be potentiated by aflibercept e.g. mucositis and diarrhea. These are often managed by FOLFIRI dose reductions or a temporary discontinuation in aflibercept treatment.

## 7. Management of Aflibercept-Related Adverse Events in My mCRC Patients—An Asian Perspective

The ESMO guidelines have been adapted into pan-Asian consensus guidelines to take into account ethnic differences in toxicity and efficacy of certain systemic treatments in patients of Asian ethnicity [50]. Both guidelines are generally aligned with only a few revisions added in the pan-Asian adaptation. Japanese Society for Cancer of the Colon and Rectum (JSCCR) guidelines for the treatment of CRC [5] have been recently updated and now include aflibercept as one of the options in the category of VEGF-targeted agents [51]. However, as with the ESMO guidelines, there is no focus on management of AEs.

In my experience, the most common AEs observed with aflibercept plus FOLFIRI are consistent with those reported in clinical trials: neutropenia, leukopenia, hypertension, diarrhea, fatigue and decreased appetite. If AEs occur due to this combination treatment, the causative agent should be identified, if possible. These toxicities can be managed using symptomatic treatment for prevention, and in the majority of cases of AEs, aflibercept treatment is continued. In rare cases, treatment may need to be changed or discontinued but, according to UPCR guidelines, this is only with the occurrence of serious (grade ≥ 3) proteinuria with inadequate treatment response, according to UPCR guidelines. Dose reductions of 5-fluorouracil should be considered in cases of grade ≥ 3 neutropenia, grade ≥ 3 fatigue and diarrhea. Reduction of aflibercept should be considered in cases of for grade ≥ 3 proteinuria.

Rarely, granulocyte-colony stimulating factor (G-CSF) is used for prophylactic and therapeutic treatment of neutropenia. Low-risk patients without fever will not receive G-CSF even if they have grade 4 neutropenia when using FOLFIRI with aflibercept. However, the use of G-CSF varies across intuitions. Antimicrobial agents should be administered in cases of febrile neutropenia.

In the management of hypertension, patients are started with a calcium antagonist; there is a paucity of evidence for effective front-line treatment with angiotensin-receptor blockers (ARBs) in this setting. Calcium channel antagonists are also considered to be effective because of their relatively strong antihypertensive effect and the mechanism of action that reduces vascular smooth muscle cell contraction caused by impaired NO signaling induced by VEGF inhibitors. We consider the use of ARBs, angiotensin-converting-enzyme (ACE) inhibitors, beta-blockers and diuretics, dependent on the patient’s condition, but most frequently use a combination of calcium antagonist plus ARB or ACE inhibitor. Hypertension could generally be improved by stopping or reducing the dose of VEGF inhibitors, since hypertension with the causative drug is reversible.

In cases of diarrhea, symptomatic treatment may be incorporated using loperamide or infusion treatment (for the worst cases of dehydration only). Prophylactic treatment with 5-hydroxytryptamine type 3 and steroids are used for the prevention of vomiting. Thus, we find that the safety profile of aflibercept is manageable; most AEs can be controlled without the need to discontinue treatment.

## 8. Discussion

Our review of the AEs reported among patients with mCRC receiving aflibercept in different populations and settings indicates that the range, incidence and severity of aflibercept-associated AEs is generally evenly distributed across geographies and in some instances appear to be less frequently reported in real-world/retrospective studies compared with clinical trials.

In the US real-world study by Ivanova and colleagues, although patients receiving aflibercept were more heavily pretreated and potentially less robust compared with the VELOUR trial, AE rates were similar to or lower than the VELOUR trial [42]. However, the lower incidence of reported AEs in the post-marketing setting may be due to underreporting as the reliance is on spontaneous reporting, which may fail to capture all occurring AEs [52].

Guidance for the management of clinically relevant AEs is available [48]. Of note, strict monitoring of blood pressure and immediate management of hypertension during therapy is mandatory [19]. In the large randomized trial of aflibercept plus FOLFIRI versus placebo plus FOLFIRI, although grade 3/4 AEs were more frequent in the aflibercept arm, they occurred in early treatment cycles and decreased sharply following initial presentation [14].

## 9. Conclusions

Aflibercept is an antiangiogenic agent that targets VEGF-A, VEGF-B and PlGF. It was approved for the treatment of patients with mCRC who had progressed on oxaliplatin following the pivotal VELOUR trial.

Data suggests that the AE profile of aflibercept combination therapy trends towards being more favorable in the real-world compared with clinical trial settings, with no new safety signals identified irrespective of geographical location. Real-world studies reported neutropenia, hypertension and gastrointestinal AEs as being frequently associated with aflibercept, similar to clinical trials. However, the rates at which these occurred was often lower in real-world studies compared with clinical trials. This may be a result of under-reporting or improved patient management. Furthermore, AEs are generally manageable and comparable across various geographic locations. As such, safety experience gained in clinical trials can translate into the clinical setting across various countries, for example, identifying when extra vigilance and close monitoring may be appropriate.

## Figures and Tables

**Table 1:** Summary of adverse events (any grade) ≥ 5% in the VELOUR trial of placebo plus FOLFIRI vs aflibercept plus FOLFIRI [11].

Adverse Event	Placebo Plus FOLFIRI (*n* = 605)			Aflibercept Plus FOLFIRI (*n* = 611)		
	All grades (%) *	Grade 3 (%) *	Grade 4 (%) *	All Grades (%) *	Grade 3 (%) *	Grade 4 (%) *
Any	97.9	45.1	17.4	99.2	62.0	21.4
Diarrhea	56.5	7.6	0.2	69.2	19.0	0.3
Asthenic conditions	50.2	10.4	0.2	60.4	16.0	0.8
Stomatitis and ulceration	34.9	5.0	—	54.8	13.6	0.2
Nausea	54	3.0	—	53.4	1.8	—
Infections and infestations	32.7	6.1	0.8	46.2	11.0	1.3
Hypertension	10.7	1.5	—	41.4	19.1	0.2
Hemorrhage	19	1.7	—	37.8	2.8	0.2
Epistaxis	7.4	—	—	27.7	0.2	—
GI and abdominal pains	29.1	3.1	0.2	34	5.1	0.3
Vomiting	33.4	3.5	—	32.9	2.6	0.2
Decreased appetite	23.8	1.7	0.2	31.9	3.4	—
Weight decreased	14.4	0.8	—	31.9	2.6	—
Alopecia	30.1	—	—	26.8	—	—
Dysphonia	3.3	—	—	25.4	0.5	—
Constipation	24.6	1.0	—	22.4	0.8	—
Headache	8.8	0.3	—	22.3	1.6	—
PPES	4.3	0.5	—	11.0	2.8	—
VTE	7.3	2.6	3.6	9.3	3.1	4.7
Anemia	91.1	3.5	0.8	82.3	3.3	0.5
Neutropenia	56.3	19.1	10.4	67.8	23.1	13.6
Thrombocytopenia	33.8	0.8	0.8	47.4	1.7	1.7
Proteinuria	40.7	1.2	—	62.2	7.5	0.3
ALT increased	37.1	2.2	—	47.3	2.5	0.2

ALT, alanine aminotransferase; GI, gastrointestinal; PPES, palmar-plantar erythrodysesthesia syndrome; VTE, venous thromboembolic event. * Grades were determined according to National Cancer Institute Common Terminology Criteria of Adverse Events, version 3.0.

**Table 2:** Safety data from the fifth interim analysis of ASQoP compared with VELOUR [15,16].

Adverse Events	ASQoP Aflibercept + FOLFIRI (*n* = 779)			VELOUR Aflibercept + FOLFIRI (*n* = 611)		
	All Grades (%) [15]	Grade 3–4 (%) [15]	Grade 4 (%) [16]	All grades (%)	Grade 3–4 (%)	Grade 4 (%)
Any treatment-emergent adverse event	98.7	78.2	18.0	99.2	83.5	21.4
Selected treatment-emergent adverse events of any grade in ≥ 20% of patients						
Diarrhea	61.6	15.3	0.3	69.2	19.3	0.3
Asthenic conditions	57.8	13.6	0	60.4	16.9	0.8
Hypertension	48.4	24.1	0	41.4	19.3	0.2
Stomatitis ^1^ (and ulcerations) ^2^	42.9	10.5	0.1	54.8	13.7	0.2
Infections and infestations ^2^	31.3	11.7	2.1	46.2	12.3	1.3
Venous thromboembolic events ^2^	6.2	4.1	0.9	9.3	7.9	4.7
Arterial thromboembolic events ^2^	2.3	0.8	0.3	2.6	1.8	1.0
Neutropenia	60.5 *	30.5 *	9.7 *	67.8 **	36.7 **	13.6 **
Proteinuria	60.1	7.6	0.6	62.2	7.9	0.3

* *n* = 744; ** *n* = 603.

**Table 3:** General guidelines for aflibercept dosing and schedule modification due to adverse events as categorized by CTCAE 4.0 * [48].

Event	Action to Be Taken
Hypertension Grade 3 Grade 4	If not controlled with medication, discontinue aflibercept Discontinue aflibercept
Proteinuria > 2 g protein/24 h Grade 4 proteinuria (nephrotic syndrome)	Hold aflibercept until proteinuria improves to < 2 g of protein/24 h Discontinue aflibercept in patients with > 2 g proteinuria/24 h that does not resolve within 3 months after holding aflibercept. Work-up for proteinuria such as renal biopsy should be considered Discontinue aflibercept
Gastrointestinal perforation Gastrointestinal perforation or dehiscence	Discontinue aflibercept
Thromboembolic events Grade 3 venous thromboembolic event or incidentally discovered pulmonary embolus first occurrence Any grade arterial thromboembolic event or symptomatic Grade 4 venous thromboembolic event first occurrence	Hold aflibercept treatment If the planned duration of therapeutic-dose anticoagulant therapy is < 2 weeks, aflibercept should be held until the period of therapeutic-dose anticoagulant therapy is over If the planned duration of therapeutic-dose anticoagulant therapy is > 2 weeks, aflibercept should be held for 2 weeks and then may be resumed during the period of therapeutic-dose anticoagulant therapy as soon as all of the following criteria are met: The patient must be on a stable dose of anticoagulant and, if on warfarin, have an INR within the target range (usually between 2 and 3) prior to restarting study drug treatment The patient has no history of Grade 3 or 4 hemorrhagic events before starting aflibercept The patient has no evidence of tumor invading or abutting major blood vessels on any prior CT scan Discontinue aflibercept
Hemorrhage Grade 1 and 2 Grade 3 or 4 (first occurrence)	No dose modification Discontinue aflibercept

* CTCAE updated to version 5; changes between version 4 and 5 include clarification to vascular hypertension, proteinuria, gastrointestinal perforation, thromboembolic events and hemorrhage grading definitions. INR, International Normalized Ratio.
